# Association of Dipeptidyl Peptidase-4 Inhibitors Use with Reduced Risk of Hepatocellular Carcinoma in Type 2 Diabetes Patients with Chronic HBV Infection

**DOI:** 10.3390/cancers15041148

**Published:** 2023-02-10

**Authors:** Tzu-I Chen, Fu-Jen Lee, Wan-Lun Hsu, Yong-Chen Chen, Mingchih Chen

**Affiliations:** 1 Graduate Institute of Business Administration, College of Management, Fu Jen Catholic University, New Taipei City 242062, Taiwan; 2 Division of Gastroenterology and Hepatology, Department of Internal Medicine, Fu Jen Catholic University Hospital, New Taipei City 24352, Taiwan; 3 Master Program of Big Data Analysis in Biomedicine, College of Medicine, Fu Jen Catholic University, New Taipei City 242062, Taiwan; 4 Data Science Center, College of Medicine, Fu Jen Catholic University, New Taipei City 242062, Taiwan; 5 Artificial Intelligence Development Center, Fu Jen Catholic University, New Taipei City 242062, Taiwan

**Keywords:** dipeptidyl peptidase-4 inhibitors, DPP-4 inhibitors, type 2 diabetes mellitus, chronic hepatitis B virus infection, hepatocellular carcinoma, antiglycemic agents

## Abstract

**Simple Summary:**

Hepatocellular carcinoma (HCC) was the sixth most common cancer and the third leading cause of cancer death worldwide in 2020. Several studies have demonstrated that both chronic hepatitis B virus (HBV) infection and type 2 diabetes mellitus (T2DM) are important risk factors for HCC. Treatment with antiglycemic agents in patients with various chronic liver diseases has been well studied, but there are relatively few related studies on dipeptidyl peptidase-4 inhibitors (DPP-4 inhibitors), especially in patients with coexisting T2DM and chronic HBV infection. This long-term retrospective population-based cohort study aimed to investigate whether the use of DPP-4 inhibitors decreases the risk of developing HCC in patients with coexisting T2DM and chronic HBV infection. Our study presents a preliminary confirmation that DPP-4 inhibitors have a beneficial effect in decreasing the risk of HCC in the treatment of T2DM patients with chronic HBV infection.

**Abstract:**

Previous studies have indicated that HBV infection and T2DM are the factors that increase the risk of developing HCC. The experimental evidence has shown that antiglycemic agents may reduce the risk of HCC. However, the effect of dipeptidyl peptidase-4 inhibitors (DPP-4 inhibitors) on the risk of HCC in T2DM patients with chronic HBV infection remains unclear. In this retrospective cohort study, we extracted patients with T2DM and chronic HBV infection from the National Health Insurance Research Database (NHIRD) in Taiwan. The cases were divided into DPP-4 inhibitors use and non-use groups, according to whether they received DPP-4 inhibitors treatment, and the risk of HCC was compared between the two groups. At the end of the follow-up, approximately 2.33% of DPP-4 inhibitors users had received an HCC diagnosis compared with 3.33% of non-DPP-4 inhibitors users (*p* < 0.0001). After multivariate adjustment, DPP-4 inhibitors users showed a significant reduction in HCC risk (adjusted hazard ratios (aHRs): 0.53; 95% confidence intervals (CIs): 0.44–0.65). In conclusion, this population-based retrospective cohort study indicated that, in T2DM patients with chronic HBV infection, the use of DPP-4 inhibitors significantly reduced the risk of developing HCC compared with non-DPP-4 inhibitors use.

## 1. Introduction

Primary liver cancer includes hepatocellular carcinoma (HCC) (comprising 75–85% of cases) and intrahepatic cholangiocarcinoma (10–15%), as well as other rare types of carcinoma. It is the sixth most common cancer and the third leading cause of cancer death worldwide in 2020, with approximately 906,000 new cases and 830,000 deaths [1]; it is the leading cause of death among those with chronic HBV infection [2,3]. Chronic HBV infection is highly prevalent and a major health problem in Taiwan and other countries worldwide [4]. Hepatitis B is a viral infection that attacks the liver and may cause chronic hepatitis, cirrhosis, hepatic decompensation, and HCC. The WHO estimates that 296 million people were living with chronic HBV infection in 2019, with 1.5 million new infections each year. In 2019, hepatitis B resulted in an estimated 820,000 deaths, the majority due to cirrhosis and HCC [5]. Taiwan is an HBV endemic area, about half of chronic HBV carriers are infected by perinatal transmission from carrier mothers to newborns [6], and the rest are infected early in life [7]. Taiwan launched a national HBV vaccination program in 1984, and the prevalence of Hepatitis B surface antigen (HBsAg) decreased from 9.8% in the pre-vaccinated period in 1984 to 0.5% in the vaccinated cohort in 2014 [8]. The rate of spontaneous HBsAg clearance is extremely low [9]. Thus, although most of chronic hepatitis B is diagnosed in adulthood, these patients have typically been infected for decades [10].

In past two decades, as a result of aging and changes in lifestyle and dietary habits, the incidence and prevalence of various chronic diseases, such as diabetes, have been increasing gradually. The global diabetes prevalence in 20–79-year-olds in 2021 was estimated to be 10.5% (536.6 million people) and is estimated to rise to 12.2% (783.2 million) in 2045. In addition, it is also estimated that over 6.7 million people aged 20–79 will die from diabetes-related causes in 2021. Global diabetes-related health expenditure was estimated at 966 billion USD in 2021, and is projected to reach 1054 billion USD by 2045 [11]. In Taiwan, where T2DM has been the fifth leading cause of death over the past decade, the total population with DM increased by 66% between 2005 and 2014, with increasing prevalence in all age groups. In the adult population (20–79 years) the age-standardized prevalence increased by 41%, from 4.57% to 6.45%, *p* < 0.001 [12]. According to the Taiwan NHI report, T2DM ranks second among the top 10 medical expenses in 2019, about 1.53 million people had medical treatment and total medical costs were NTD 30.8 billion. The treatment of T2DM patients and the consequences of other diseases or complications arising from T2DM has become a huge economic burden in Taiwan and worldwide.

Recently more and more studies have shown that T2DM in adulthood and chronic HBV infection are significant risk factors for HCC [13,14]. Although there are no population-based studies estimating the prevalence of co-existing T2DM and chronic HBV infection in Taiwan, one hospital-based case-control study estimated the prevalence at approximately 14.5% [15]. Previous studies have indicated that insulin resistance (IR) was a significant factor associated with HCC development in chronic HBV infected patients [16], while T2DM is associated with the increased risk of a spectrum of cancers and there is an associate relationship between HbA1c and HCC risk, with aHRs of 1.37 (95% CIs: 1.08–1.74) [17]. Over the last decade, evidence has demonstrated that patients with T2DM are more likely to have an increased risk of progression from steatosis to non-alcoholic steatohepatitis (NASH) to advanced fibrosis, including cirrhosis and HCC [18,19]. Meta-analysis suggests that T2DM may be associated with elevated risks of both HCC incidence and mortality [3]. Another meta-analysis study reported that T2DM was significantly associated with the increased risk of HCC among patients with chronic hepatitis B virus infection: the random-effects HR was 1.36 (95% CIs: 1.23–1.49) [20].

Earlier observation studies have indicated that hepatitis virus infection and T2DM are risk factors for HCC and meta-analysis studies have shown that several antiglycemic agents are able to prevent HCC [21,22,23]. In the past decade, more and more antiglycemic agents with different mechanisms have been used clinically. In contrast to type 1 diabetes, which is treated only with insulin, drugs with different mechanisms have been developed for T2DM treatment, including biguanides (BG), sulfonylureas (SU), meglitinides (MG), thiazolidinediones (TZD), alpha-glucosidase inhibitors (AGI), dipeptidyl peptidase-4 inhibitors (DPP-4 inhibitors), glucagon-like peptide-1 (GLP-1) agonists, sodium glucose co-transporter-2 inhibitors (SGLT-2), etc. [24]. However, the long-term outcomes of using antiglycemic drugs in patients with chronic liver disease have yet to be uncovered.

Both GLP-1 and glucose-dependent insulinotropic polypeptide (GIP) can stimulate pancreatic beta cells to secrete insulin in a glucose-dependent manner to control blood glucose levels, with hypoglycemia occurring very rarely as an adverse effect [25]. The blocking of DPP-4, which is required for degrading GLP-1 and GIP, with DPP-4 inhibitors prolongs the activity of GLP-1 and GIP. Hepatic metabolism is a minor pathway for DPP-4 inhibitors, and a major part of the administered drug is either excreted unchanged by the renal pathway or through hydrolysis by multiple tissues/organs [26]. DPP-4 inhibitors can increase GLP-1 and GIP levels in splanchnic and portal circulations, which can promote nitro oxide production, accelerate portal vein inflow, and normalize arterial hypocontractility [27,28]. DPP-4 inhibitors may offer the prevention of further metabolic deterioration, especially in chronic liver disease or hepatic impairment patients. Taiwan NHI began reimbursing the cost of DPP-4 inhibitors in 2009 and the usage of DPP-4 inhibitors then increased quickly with time, from 3.73% in 2009 to 19.64% in 2012. Several studies have focused on reducing the risk of HCC in patients with varying degrees of chronic liver disease [23,29], nonalcoholic fatty liver disease (NAFLD) [22], or chronic HCV infection treated with antiglycemic drugs [30]. Although one study showed that thiazolidinediones reduce the risk of hepatocellular carcinoma and hepatic events in diabetic patients with chronic hepatitis B infection [31], there are relatively few on DPP-4 inhibitors in this group patient.

There was a study conducted by Taiwan NHIRD to investigate whether the use of DPP-4 inhibitors is associated with a decreased risk of HCC in patients with T2DM and chronic HCV infection. The findings indicated that the use of DPP-4 inhibitors was associated with a lower risk of HCC in patients with T2DM and chronic HCV infection [30]. However, the influences of the presence of DPP-4 inhibitors and other comorbidities on HCC risk in T2DM patients with chronic HBV infection have not been investigated. In this study, we used a nationwide database to explore whether DPP4 inhibitors use decreases the risk of developing HCC in T2DM patient with chronic HBV infection.

## 2. Materials and Methods

### 2.1. Data Resource

We conducted this nationwide population-based retrospective cohort study by using insurance claim data from the Taiwan National Health Insurance Research Database (NHIRD). The Taiwan National Health Insurance (NHI) program was launched in March of 1995 and enrollment is more that 99% of all 23.7 million residents of Taiwan. The NHIRD contains detailed claims information on International Classification of Disease codes, drug prescriptions, medical procedures, outpatient visits, inpatient hospitalizations, dates, and so on. In this study, we obtained diagnosis and treatment information through the NHIRD and newly diagnosed HCC was identified using the Taiwan Cancer Registry. The death certification profile was used to determine if any cases in our cohort died during the follow-up period and the death date was recorded.

### 2.2. Study Population

**Figure 1:**
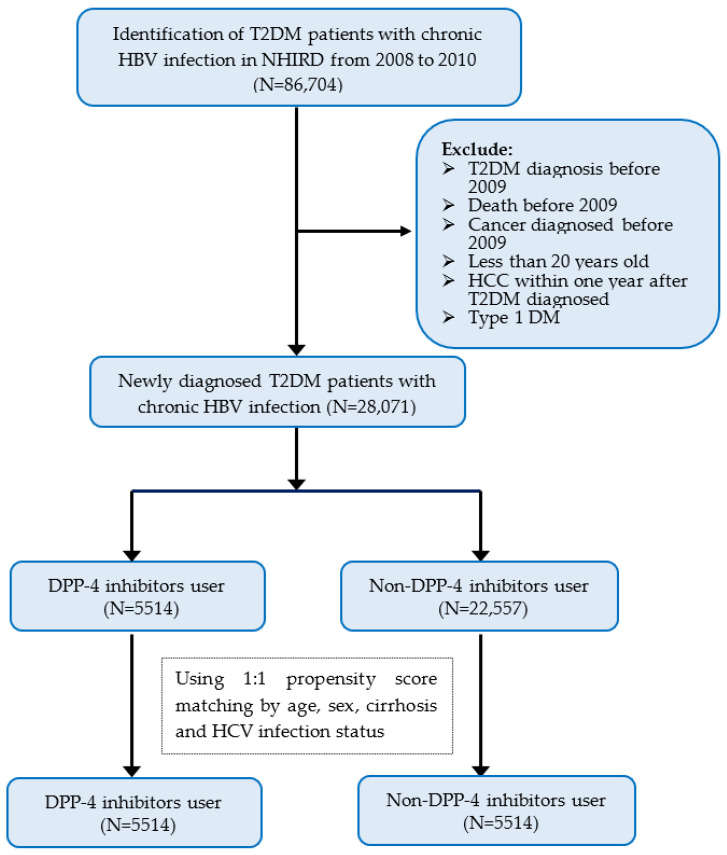
Flow chart of study patient selection. Abbreviations: T2DM, type 2 diabetes mellitus; HBV, hepatitis B virus infection; NHIRD, National Health Insurance Research Database; DPP-4 inhibitors, dipeptidyl peptidase-4 inhibitors.

This retrospective cohort study used the Taiwan NHIRD from 1 January 2008 to 31 December 2016. For the period 2008 to 2010, diagnosed T2DM patients with chronic HBV infection and aged ≥20 were selected. Only those patients diagnosed with T2DM with at least one hospital admission code with the diagnosis or with ≥3 outpatient visits were included to ensure diagnostic accuracy [32,33]. Exclusion criteria were patients diagnosed with T2DM, cancers, or death before 1 January 2009 or the occurrence of HCC within one year of T2DM diagnosis (Figure 1).

### 2.3. Definition of DPP4 Inhibitors Use and Comorbidities

The cost of DPP4 inhibitors for the treatment of T2DM began to be reimbursed by NHI in March, 2009 and the prescriptions included sitagliptin, vildagliptin, saxagliptin, alogliptin, and linagliptin. The anatomical Therapeutic Chemical (ATC) code in the patients’ post-T2DM-diagnosis prescription records, oral antiviral drug use, and insulin use are shown in Supplementary Table S1. Chronic HBV or HCV infection, and the comorbidities of cirrhosis were identified using International Classification of Diseases, Ninth Revision or Tenth Revision (ICD-9 or ICD-10, respectively) codes (Supplementary Table S2).

### 2.4. Follow-Up to Newly Diagnosed HCC

Patients were followed from the date of T2DM diagnosis until 31 December 2016. HCC incidence and dates of diagnosis were determined using ICD-9 code 155 and ICD-10 code C22 after data linkage with the National Cancer Registry and death profile. In all cases, the diagnosis of HCC was obtained from cancer registry based on pathological and imaging criteria. The percentage of morphological verification was 93.0% for all sites combined and 97.6% for all sites, excluding the liver [34]. According to the Cancer Registry Annual Report 2020, 54.53% of liver cancers were confirmed by cytology or histopathology [35]. The follow-up duration for each patient was calculated as the time from the T2DM diagnosis date to the date of HCC diagnosis, the date of death, or the end of the study period (i.e., 31 December 2016), whichever came first.

### 2.5. Study Design

The study population consisted of DPP-4 inhibitors use and non-DPP-4 inhibitors use groups, both selected from the NHIRD. In order to minimize the potential confounding effects of an imbalanced distribution of variables between DPP-4 inhibitors use and non-DPP-4 inhibitors use groups, we used the propensity score matching (PSM) method to balance the observed baseline characteristics between these two groups. Using these covariates, including age, sex, chronic HCV infection, and cirrhosis, the two groups were matched in a 1:1 ratio in accordance with recommendations from previous literature [36]. The study outcome was the occurrence of HCC in both comparison groups.

### 2.6. Statistical Analysis

Continuous data for all baseline characteristics were summarized as mean ± SE of the mean, and categorical data were presented with exact numbers and proportions. A Chi-square test and Student’s *t*-test were used to compare the difference between DPP-4 inhibitor use and non-DPP-4 inhibitor use group. The incidence of HCC was calculated by dividing the number of cases by the number of person-years followed. Cumulative incidences of study outcomes were estimated by the Kaplan–Meier method and the log-rank test for the comparison of difference between two groups. Cox proportional hazards models were used to obtain crude and adjusted HRs with 95% CIs for the effect of DPP-4 inhibitor use on HCC risk. All baseline characteristics were analyzed in univariate, and significant covariates in univariate models were further included in multivariate Cox regression models to examine associations with incident HCC. Two-tailed tests were used for testing statistical significance and a *p* value of <0.05 was considered statistically significant. All statistical analyses were performed using SAS version 9.4 (SAS Institute, Cary, NC, USA).

## 3. Results

### 3.1. Baseline Characteristics

A total 28,071 cases were included in our study, consisting of 15,384 females and 16,459 males with T2DM and chronic HBV infection. Table 1 shows the demographic and clinical characteristics for the DPP-4 inhibitors use and non-DPP-4 inhibitors use groups before and after propensity score matching. Before propensity score matching, the mean age of the DPP-4 inhibitors use group tended to be younger than that of the non-DPP-4 inhibitors use group (51.53 ± 11.24 and 52.65 ± 12.32; *p* < 0.0001) and the DPP-4 inhibitors use group contained 65.80% males and 34.20% females. In the study population, 9.67% of DPP-4 inhibitors users and 8.50% of non-DPP-4 inhibitors users received a diagnosis of HCV coinfection (*p* < 0.0059), and DPP-4 inhibitors users had a higher prevalence of cirrhosis (14.85% vs. 12.35%; *p* < 0.0001), and more frequently used anti-viral drugs (15.92% vs. 11.92%; *p* < 0.0001) and insulin (27.89% vs. 5.71%; *p* < 0.0001) compared with non-DPP-4 inhibitors users; there were fewer deaths (7.22% vs. 10.60%; *p* < 0.0001) among DPP-4 inhibitors users.

### 3.2. Incidence of HCC

**Figure 2:**
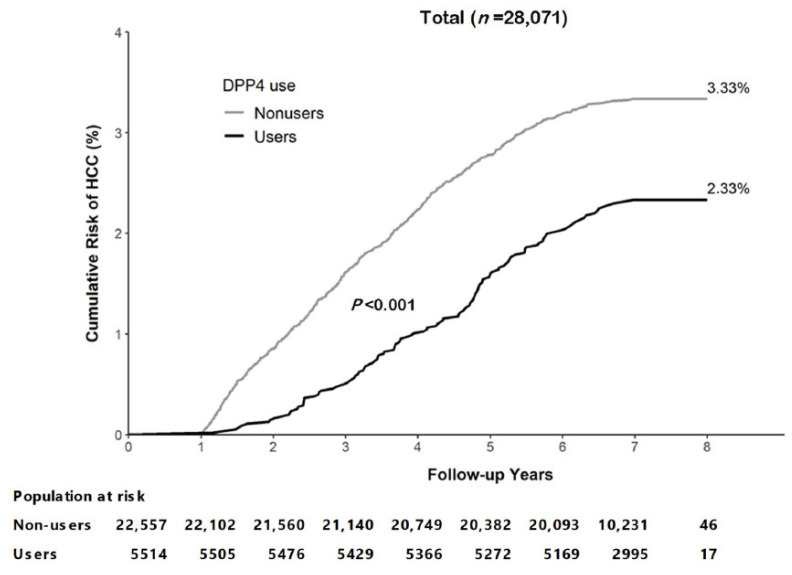
Cumulative risk of hepatocellular carcinoma (HCC) in T2DM patients with chronic HBV infection with and without DPP-4 inhibitors use.

After 28,071 person-years of follow-up, 832 cases of HCC were identified; the HCC incidence was lower among DPP-4 inhibitors users than non-DPP-4 inhibitors users (323.31 and 480.69 per 100,000 person-years, respectively). After propensity score matching, DPP-4 inhibitors users had lower HCC incidence than non-DPP-4 inhibitors users (323.31 and 532.59 per 100,000 person-years, respectively). Patients with advanced age, male sex, cirrhosis, oral anti-virus drug use, insulin use, and who acquired HCV coinfection had a higher HCC incidence, whether before or after propensity score matching (*p* < 0.0001; Table 2). At the end of the follow-up, approximately 2.33% of DPP-4 inhibitors users had received an HCC diagnosis compared with 3.33% of non-DPP-4 inhibitors users (*p* < 0.0001; Figure 2).

### 3.3. Relative Risk of HCC with and without Treatment with DPP-4 Inhibitors

**Figure 3:**
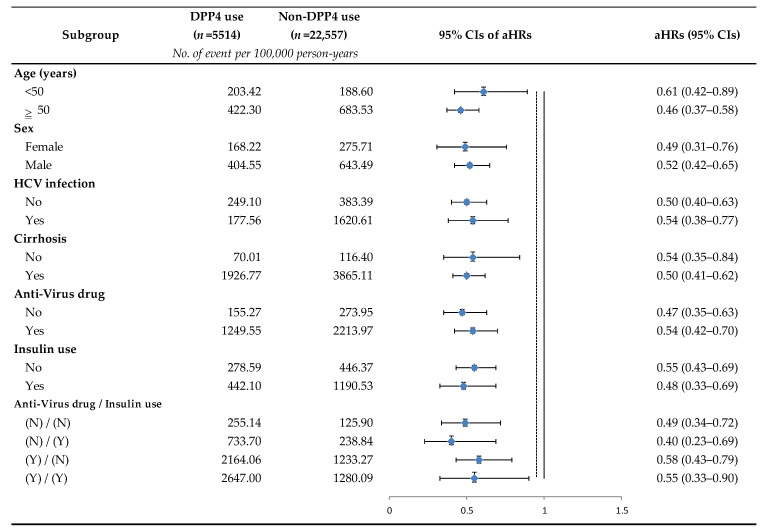
Subgroup analysis of DPP-4 inhibitors use for the reduced risk of hepatocellular carcinoma. Stratified by one of the following parameters and adjusted for the remaining of the following parameters: age, sex, chronic HCV infection, cirrhosis, anti-virus drug use, and insulin use. Abbreviations: HCV, hepatitis C virus; aHRs, adjusted hazard ratios; CIs, confidence intervals.

DPP-4 inhibitors users exhibited a significantly reduced risk of HCC (aHRs: 0.53, 95% CIs: 0.44–0.65) after adjustment for age, sex, chronic hepatitis C virus infection, cirrhosis, anti-virus drug use, and insulin use (Table 3). After propensity score matching, in DPP-4 inhibitors users, adjusted for the aforementioned covariates, the risk of HCC was consistently reduced (aHRs: 0.53, 95% CIs: 0.42–0.68). In multivariate adjusted models, advanced age, HCV coinfection, anti-virus drug use, and cirrhosis were associated with a significant risk of HCC (*p* < 0.0001). In the subgroup analysis (Figure 3), the use of DPP-4 inhibitors was shown to be beneficial in the prevention of HCC, and the incidence of HCC was generally lower among the subgroups within DPP-4 inhibitors users compared within non-DPP-4 inhibitors users. The effect of DPP-4 inhibitors use group on lower HCC risk consistently favored DPP-4 inhibitors users across many prespecified subgroups.

## 4. Discussion

Our study demonstrated that the use of DPP-4 inhibitors in T2DM patients with chronic HBV infection can significantly decrease the risk of HCC (aHRs: 0.53, 95% CIs: 0.44–0.65). To the best of our knowledge, our study is the first large population-based cohort study investigating the long-term outcomes associated with HCC risk in T2DM and chronic HBV infection patients using DPP-4 inhibitors. We followed 185,539 person-years of T2DM patients with chronic HBV infection and obtained 832 HCC cases over 8 years. A study conducted using the Longitudinal Health Insurance Database 2000, a subset of NHIRD, including a representative sample of one million randomly drawn from all enrollees in the National Health Insurance program registry in 2000, indicated that T2DM patients with chronic HCV infection using DPP-4 inhibitors had a lower risk of HCC (aHRs: 0.59, 95% CIs: 0.43–0.79) [30]. Our study was conducted using population-based data and showed that treatment with DPP-4 inhibitors was similarly protective in T2DM patients with chronic HBV infection. DPP-4 inhibitors reduced TNFα or LPS-induced cellular reactive oxygen species (ROS) levels, cell apoptosis, and protein expression in the NFκB signaling pathway in HepG2 cells or primary mouse hepatocytes. It may protect liver tissue by alleviating ROS production and NF-κB signaling activation, thus providing a putative mechanism to prevent the development of diabetic liver disease [37]. In an animal study, DPP4 inhibitors suppressed HCC development by activating lymphocyte infiltration into xenograft tumors or liver tumors in mice. This effect was exerted by preventing the biologically active form of CXCL10 from being truncated by DPP4 activity [38]. Although it is unclear whether DPP-4 inhibitors are directly involved in the regression of HCC, the marked invasion of CD8+ T-cells was seen around the HCC tissue, suggesting that the DPP-4 inhibitors may improve the immune response [39]. According to those studies, DPP-4 inhibitors may lower the HCC risk in patients with coexisting DM and chronic HBV infection in the same way. The possible mechanisms of DPP-4 inhibitors and HCC are found in Supplementary Figure S1.

Furthermore, T2DM is closely associated with non-alcoholic fatty liver disease (NAFLD) and with the faster progression of NAFLD to NASH, cirrhosis, and HCC. Increasing evidence suggests that newer classes of antiglycemic agents, such as peroxisome proliferator-activated receptor agonists (PPAR), GLP-1 agonists, DPP-4 inhibitors, or SGLT-2 inhibitors, could reduce the rates of NAFLD progression [40,41,42]. Our study suggests that these additional extra-hepatic benefits of DPP-4 inhibitors are potentially important, not only in NAFLD, but also for T2DM patients with chronic hepatitis virus infection.

In the subgroup analysis (Figure 3), the risk of HCC appeared to be reduced more by using DPP-4 inhibitors in the older patient group (≥50 years old) than in the younger group (<50 years old). Previous studies have shown that, compared to patients aged 30–39 years with chronic HBV infection, those aged 50–59 and ≥60 have higher seroclearance ratios (aHRs: 1.42; 95% CIs: 1.14–1.77 and aHRs: 2.13; 95% CIs: 1.62–2.81, respectively) [9]. This may indicate that viral loads will decrease over time, and we speculate that older patients may respond better to the HCC protective effects of DPP-4 inhibitors.

Our study has several strengths. We recruited patients from the NHIRD, which covers approximately 99% of the population of Taiwan. This population-based cohort study of T2DM patients with chronic HBV infection could demonstrate the long-term effects of DPP-4 inhibitors on HCC. In our cohort, we included patients with newly diagnosed diabetes in 2009–2010, when the Taiwan NHI began to reimburse the cost of DPP-4 inhibitors (2009, 2011, 2011, 2012, and 2015 for sitagliptin, saxagliptin, vildagliptin, linagliptin, and alogliptin, respectively), that accessed the treatment concurrently. Events recorded within one year after the index-date were excluded to reduce the possibility of latent HCC incidence; despite this precaution, it was revealed that DPP-4 inhibitors could significantly lower the risk of HCC. We compared the data from the study cohort before and after propensity score matching to understand the effects of using DPP-4 inhibitors. As the result, the group using DPP-4 inhibitors consistently showed a reduced risk of HCC before and after propensity score matching. Although the risk did not become lower after matching, we obtained a narrower confidence interval for the results. We adjusted for patients who had ever used anti-virus drugs or insulin, as previous studies suggested these might reduce the risk of HCC. Subgroup analysis showed that patients in the DPP-4 inhibitors use group still had the positive benefits, whether or not they received the anti-virus drug or insulin therapy.

### The Limitations of Our Study

Our study has some limitations. First, the claims data lacked some patient information, such as body mass index, family history, lifestyle, blood test results, and HbA1c levels that could have affected the measurement of the outcomes. Since Taiwan National Insurance provides comprehensive T2DM treatment, most patients are likely to have good drug compliance. Although the lack of HbA1c levels was one of the limitations of our study, the results tended to estimate the protective effect of DPP-4 inhibitors more conservatively. Second, although NIH medical records provided accurate prescription information, we were unable to assess patient adherence to medication, which may have led us to underestimate drug efficacy. Third, we were not able to obtain the exact date of chronic HBV infection, nor could we restrict T2DM diagnosed before or after chronic HBV infection in this study. Since perinatal or early postnatal transmission is the most important source of chronic HBV infection in Taiwan [43], patients may be unaware of their infection status and be diagnosed only when any relevant symptoms or clinical needs arise. Finally, DPP-4 inhibitors are used as the second-line treatment in Taiwan; according to the treatment guidelines, patient are prescribed metformin as the first-line treatment, and other antiglycemic agents are changed or added depending on HbA1c control or the patient’s conditions. Since Taiwan NIH reimburses the cost of many available antiglycemic agents, patients may have different prescription combinations, which may have overestimated the effects of DPP-4 inhibitors in our study. This study shows that the use of DPP-4 inhibitors can reduce the risk of HCC, and further research could investigate some major antiglycemic agents in combination with DPP-4 inhibitors to clarify the impact of different antiglycemic agents on the risk of HCC. Our retrospective observational study relied on insurance claims data and the findings must be interpreted with caution, as claims data may be incomplete and contain unknown confounding factors due to inherent limitations in purpose and design.

## 5. Conclusions

In conclusion, this large nationwide retrospective study indicated that, among T2DM patients with chronic HBV infection, the use of DPP-4 inhibitors was associated with a significantly lower risk of HCC incidence than non-DPP-4 inhibitors use. It was preliminarily confirmed that DPP-4 inhibitors have a beneficial effect on reducing the risk of HCC in T2DM patients with chronic HBV infection. The results of this study provide treatment recommendations for T2DM patients, especially those with chronic HBV infection. In the future, more prospective studies should be carried out to observe liver-specific endpoints after the use of DPP-4 inhibitors or different antiglycemic agents in combination with DPP-4 inhibitors in T2DM patients with hepatitis virus infection.

## Figures and Tables

**Table 1:** Baseline characteristics of the study population, stratified by the use of DPP-4 inhibitors before and after propensity score match.

Characteristics	Before Propensity Score Matching			After Propensity Score Matching		
	DPP-4 Inhibitors Use	Non-Use	*p* Value	DPP-4 Inhibitors Use	Non-Use	*p* Value
	(N = 5514)	(N = 22,557)		(N = 5514)	(N = 5514)	
Age (Years)						
20–29	780 (14.15)	3388 (15.02)	<0.0001	780 (14.15)	779 (14.13)	-
30–39	1679 (30.45)	5571 (24.70)		1679 (30.45)	1679 (30.45)	
40–49	1789 (32.44)	7365 (32.65)		1789 (32.44)	1789 (32.44)	
50–59	904 (16.39)	4061 (18.00)		904 (16.39)	905 (16.41)	
60+	362 (6.57)	2172 (9.63)		362 (6.57)	362 (6.57)	
Mean ± SD	51.53 ± 11.24	52.65 ± 12.32	<0.0001	51.53 ± 11.24	51.50 ± 11.51	-
Sex						
Female	1886 (34.20)	9726 (43.12)	<0.0001	1886 (34.20)	1886 (34.20)	-
Male	3628 (65.80)	12,831 (56.88)		3628 (65.80)	3628 (65.80)	
HCV infection						
No	4981 (90.33)	20,640 (91.50)	0.0059	4981 (90.33)	4981 (90.33)	-
Yes	533 (9.67)	1917 (8.50)		533 (9.67)	533 (9.67)	
Cirrhosis						
No	4695 (85.15)	19,772 (87.65)	<0.0001	4695 (85.15)	4695 (85.15)	-
Yes	819 (14.85)	2785 (12.35)		819 (14.85)	819 (14.85)	
Anti-virus drug						
No	4636 (84.08)	19,867 (88.07)	<0.0001	4636 (84.08)	4770 (86.51)	0.0003
Yes	878 (15.92)	2690 (11.92)		878 (15.92)	744 (13.49)	
Insulin use						
No	3976 (72.11)	21,270 (94.29)	<0.0001	3976 (72.11)	5160 (93.58)	<0.0001
Yes	1538 (27.89)	1287 (5.71)		1538 (27.89)	354 (6.42)	
HCC						
No	5391 (97.77)	21,848 (96.86)	0.0003	5391 (97.77)	5322 (96.52)	<0.0001
Yes	123 (2.23)	709 (3.14)		123 (2.23)	192 (3.48)	
Death						
No	5116 (92.78)	20,167 (89.40)	<0.0001	5116 (92.78)	4910 (89.05)	<0.0001
Yes	398 (7.22)	2390 (10.60)		398 (7.22)	604 (10.95)	

**Table 2:** Numbers of subjects and incidence of hepatocellular carcinoma by baseline characteristics before and after propensity score match.

Baseline Characteristics	Before Propensity Score Matching					After Propensity Score Matching				
	Number of Subjects (N = 28,071)	Number of HCC (N = 832)	Person-Years of Follow-Up	Incidence Rate †	*p* Value	Number of Subjects (N = 11,028)	Number of HCC (N = 315)	Person-Years of Follow-Up	Incidence Rate †	*p* Value
DPP-4 inhibitors										
No	22,557	709	147,495	480.69	0.0003	5514	192	36,050	532.59	<0.0001
Yes	5514	123	38,044	323.31		5514	123	38,044	323.31	
Age (Years)										
20–29	4168	21	28,598	73.43	<0.0001	1559	10	10,728	93.21	<0.0001
30–39	7250	128	49,054	260.94		3358	74	22,808	324.45	
40–49	9154	287	61,187	469.05		3578	118	24,126	489.10	
50–59	4965	254	32,220	788.33		1809	80	12,037	664.62	
60+	2534	142	14,480	980.66		724	33	4394	751.02	
Sex										
Female	11,612	202	78,365	257.77	<0.0001	3772	58	25,780	224.98	<0.0001
Male	16,459	630	107,174	587.83		7256	257	48,313	531.95	
HCV infection										
No	25,621	607	170,419	356.18	<0.0001	9962	221	67,355	328.11	<0.0001
Yes	2450	225	15,119	1488.19		1066	94	6739	1394.87	
Cirrhosis										
No	24,467	178	166,015	107.22	<0.0001	9390	60	64,571	92.92	<0.0001
Yes	3604	654	19,523	3349.89		1638	255	9523	2677.73	
Anti-virus drug										
No	24,503	411	163,978	250.64	<0.0001	9406	140	63,909	219.06	<0.0001
Yes	3568	421	21,561	1952.60		1622	175	10,185	1718.21	
Insulin										
No	25,246	705	168,330	418.82	<0.0001	9136	243	61,845	392.92	<0.0001
Yes	2825	127	17,209	737.99		1892	72	12,249	587.80	

† per 100,000 person-years.

**Table 3:** DPP4 inhibitors use associated with decreased risk for hepatocellular carcinoma.

Characteristics	Before Propensity Score Matching		After Propensity Score Matching	
	Crude HR (95% CI)	Adjusted HR * (95% CI)	Crude HR (95% CI)	Adjusted HR * (95% CI)
DPP-4 inhibitors				
No	Ref	Ref	Ref	Ref
Yes	0.68 (0.56–0.82)	0.53 (0.44–0.65)	0.61 (0.49–0.76)	0.53 (0.42–0.68)
Age (Years)				
20–29	Ref	Ref	Ref	Ref
30–39	3.55 (2.24–5.63)	2.43 (1.53–3.86)	3.48 (1.80–6.74)	2.76 (1.42–5.34)
40–49	6.38 (4.09–9.93)	4.01 (2.57–6.25)	5.26 (2.76–10.02)	3.90 (2.04–7.46)
50–59	10.72 (6.87–16.72)	5.69 (3.64–8.91)	7.15 (3.71–13.80)	5.26 (2.71–10.20)
60+	13.25 (8.38–20.95)	7.24 (4.56–11.50)	8.04 (3.96–16.31)	7.32 (3.58–14.96)
Sex				
Female	Ref	Ref	Ref	Ref
Male	2.28(1.95–2.67)	1.88 (1.60–2.22)	2.37 (1.78–3.15)	2.13 (1.59–2.85)
HCV infection				
No	Ref	Ref	Ref	Ref
Yes	4.18 (3.59–4.87)	2.11 (1.79–2.48)	4.26 (3.35–5.43)	2.25 (1.74–2.92)
Cirrhosis				
No	Ref	Ref	Ref	Ref
Yes	31.51 (26.70–37.20)	16.82 (13.97–20.26)	29.08 (21.95–38.54)	15.78 (11.53–21.59)
Anti-virus drug				
No	Ref	Ref	Ref	Ref
Yes	7.80 (6.81–8.94)	2.37 (2.03–2.76)	7.87 (6.30–9.83)	2.61 (2.03–3.35)
Insulin use				
No	Ref	Ref	Ref	Ref
Yes	1.78 (1.47–2.15)	0.88 (0.72–1.07)	1.51 (1.16–1.96)	0.86 (0.65–1.14)

* adjusted for age, sex, chronic hepatitis C virus infection, cirrhosis, anti-virus drug use, insulin use. Cox’s proportional hazards models were used.

## Data Availability

The datasets generated and/or analyzed during the current study are not publicly available in accordance with the policy of the Health and Welfare Data Science Center, Ministry of Health and Welfare, Taiwan, but are available from the corresponding author upon reasonable request.

## Supplementary Materials

The following supporting information can be downloaded at: https://www.mdpi.com/article/10.3390/cancers15041148/s1, Table S1: Anatomical Therapeutic Chemical classification codes for reimbursable DPP-4 inhibitors, nucleoside and nucleotide reverse transcriptase inhibitors, and insulin in Taiwan; Table S2: ICD codes for the diagnosis of comorbidities; Figure S1: Mechanisms of action and management of DPP-4-related liver disease and refer to Sharma, A., et al. (2022) [39].
