# Supplementary material for: Association of Dipeptidyl Peptidase-4 Inhibitors Use with Reduced Risk of Hepatocellular Carcinoma in Type 2 Diabetes Patients with Chronic HBV Infection
Source: Cancers (Basel). 2023 Feb 10;15(4):1148. doi: 10.3390/cancers15041148 (PMC9954498; doi:10.3390/cancers15041148)
Supplement: Supplementary file 1 [file cancers-15-01148-s001.zip › cancers-2179855-supplementary.pdf]

Supplementary Materials: Association of Dipeptidyl peptidase-4 inhibitors use with reduced risk of Hepatocellular Carcinoma in Type 2 Diabetes Patients with chronic HBV Infection

Tzu-I Chen, Fu-Jen Lee, Wan-Lun Hsu, Yong-Chen Chen and Mingchih Chen

Table S1. Anatomical Therapeutic Chemical classification codes for reimbursable DPP-4 inhibitors, nucleoside and nucleotide reverse transcriptase inhibitors, and insulin in Taiwan

Name	ATC code
**Dipeptidyl peptidase 4 (DPP-4) inhibitors**	
sitagliptin	A10BH01
vildagliptin	A10BH02
saxagliptin	A10BH03
alogliptin	A10BH04
linagliptin	A10BH05
metformin and sitagliptin	A10BD07
metformin and vildagliptin	A10BD08
pioglitazone and alogliptin	A10BD09
metformin and saxagliptin	A10BD10
metformin and linagliptin	A10BD11
metformin and alogliptin	A10BD13
**Nucleoside and nucleotide reverse transcriptase inhibitors**	
zidovudine	J05AF01
didanosine	J05AF02
stavudine	J05AF04
lamivudine	J05AF05
abacavir	J05AF06
tenofovir disoproxil	J05AF07
adefovir dipivoxil	J05AF08
entecavir	J05AF10
telbivudine	J05AF11
tenofovir alafenamide	J05AF13
**Insulins and analogues**	
insulin (human)	A10AB01
insulin (pork)	A10AB03
insulin lispro	A10AB04
insulin aspart	A10AB05
insulin glulisine	A10AB06
combinations	A10AB30
insulin (human)	A10AC01
insulin (pork)	A10AC03

combinations	A10AC30
insulin (human)	A10AD01
insulin (pork)	A10AD03
insulin lispro	A10AD04
insulin aspart	A10AD05
insulin degludec and insulin aspart	A10AD06
insulin (human)	A10AE01
insulin glargine	A10AE04
insulin detemir	A10AE05
insulin degludec	A10AE06
insulin glargine and lixisenatide	A10AE54

Table S2. ICD codes for the diagnosis of comorbidities

Disease	ICD-9	ICD-10
Type 2 Diabetes	250.XX	E11
Chronic HBV infection	070.2, 070.3, V02.61	B18.0, B18.1, B19.1
Chronic HCV infection	070.41, 070.44, 070.51, 070.54, 070.70, 070.71, V02.62	B18.2, B19.2
Cirrhosis	571.5	K74.0, K74.1, K74.2, K74.6

Abbreviations: HBV, hepatitis B virus; HCV, hepatitis C virus; ICD-9, International Classification of Diseases, Ninth Revision; ICD-10, International Classification of Diseases, Tenth Revision.

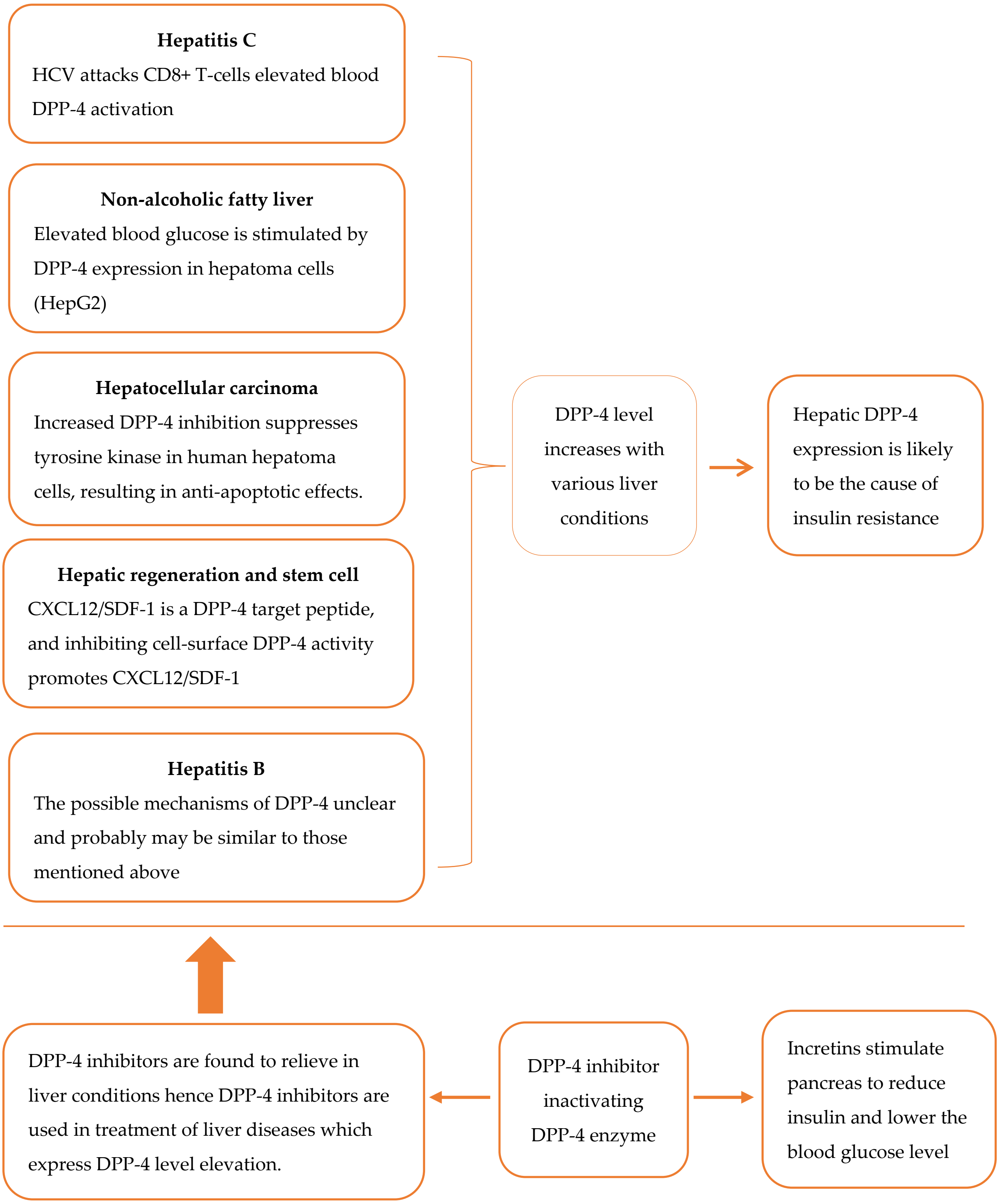

Figure S1. Mechanisms of action and management of DPP-4-related liver disease and refer to Sharma, A., et al. (2022) [39].
